# Involvement of Acylated Homoserine Lactones (AHLs) of *Aeromonas sobria* in Spoilage of Refrigerated Turbot (*Scophthalmus maximus* L.)

**DOI:** 10.3390/s16071083

**Published:** 2016-07-13

**Authors:** Tingting Li, Fangchao Cui, Fengling Bai, Guohua Zhao, Jianrong Li

**Affiliations:** 1 College of Food Science, Southwest University, Chongqing 400715, China; jwltt@dlnu.edu.cn (T.L.); dotanicepl@163.com (G.Z.); 2 College of Life Science, Dalian Nationalities University, Dalian 116029, China; 3 College of Food Science and Technology, Bohai University, Jinzhou 121013, China; cfc1031@163.com (F.C.); myan9102@163.com (F.B.)

**Keywords:** *Aeromonas sobria*, siderophore, quorum sensing, spoilage, turbot

## Abstract

One quorum sensing strain was isolated from spoiled turbot. The species was determined by 16S rRNA gene analysis and classical tests, named *Aeromonas sobria* AS7. Quorum-sensing (QS) signals (*N*-acyl homoserine lactones (AHLs)) were detected by report strains and their structures were further determined by GC-MS. The activity changes of AHLs on strain growth stage as well as the influence of different culture conditions on secretion activity of AHLs were studied by the punch method. The result indicated that strain AS7 could induce report strains to produce typical phenotypic response. *N*-butanoyl-dl-homoserine lactone (C_4_–HSL), *N*-hexanoyl-dl-homoserine lactone (C_6_–HSL), *N*-octanoyl-dl-homoserine lactone (C_8_–HSL), *N*-decanoyl-dl-homoserine lactone (C_10_–HSL), *N*-dodecanoyl-dl-homoserine lactone (C_12_–HSL) could be detected. The activities of AHLs were density-dependent and the max secretion level was at pH 8, sucrose culture, 1% NaCl and 32 h, respectively. The production of siderophore in strain AS7 was regulated by exogenous C_8_–HSL, rather than C_6_–HSL. Exogenous C_4_–HSL and C_8_–HSL accelerated the growth rate and population density of AS7 in turbot samples under refrigerated storage. However, according to the total viable counts and total volatile basic nitrogen (TVB-N) values of the fish samples, exogenous C_6_–HSL did not cause spoilage of the turbot fillets. In conclusion, our results suggested that QS was involved in the spoilage of refrigerated turbot.

## 1. Introduction

*Scophthalmus maximus* L. is one of the most important farmed fish species in China, Chile and several countries of Europe. Fish are highly perishable products, the spoilage caused by the various biochemical changes naturally occurring as well as due to microbial activities. Microbial spoilage is the most common cause of fish spoilage. Thus, microbial activity is considered to be very important for the manifestation of spoilage. In recent years, the detection of quorum sensing signals in putrid food products has added a new dimension to study the process of food spoilage.

Quorum sensing (QS), a term introduced by Fuqua Winans and Greenberg in 1994 to describe cell-to-cell communication, is a system that allowed bacteria to monitor their population density and control a number of physiological functions by releasing and receiving of signal molecules, called autoinducers (AIs) [1]. *N*-acyl-homoserine lactones (AHLs) are the most common AIs and are generally specific to Gram-negative bacteria [2]. The regulation of phenotypes such as swarming, virulence and biofilm formation, is dependent on population density, which is synchronized by the signal molecules [3,4,5,6]. The most important study of QS focused on the AHL regulating role in bacterial pathogenesis [2,7]. To date, food spoilage via microbial regulation by QS, an event leading to severe economic losses as well as public health problems, receives more attention from researchers. Some studies have identified biofilm formation regulated by QS in *Aeromonas hydrophila, Pseudomonas fluorescens* and *Serratia liquefaciens* [8,9].

The detection of AHLs is a key procedure in the QS system. By typical phenotypic response, such as β-galactosidase activity, bioluminescence, or violacin production, report stains are generally used to screen for AHL production [10,11] or in synchronous combination [12]. Each induced strain responds to a different scope of AHLs, but the results may be false positive. Thin-layer chromatography (TLC) is a fast and cheap method to detect the types of AHLs by covering the monitoring bacteria with plates and separating the extracts of bacteria as well as AHL standards [13]. Now, the detection of AHLs is more sensitive and reliable under the different circumstances. High-performance liquid chromatography-(tandem) mass-spectrometry (HPLC-(MS)/MS) and gas chromatography-mass spectrometry (GC-MS) are utilized for qualitative and quantitative analysis. LC-MS/MS applied for quantitative AHLs was validated by Ortori [14]. Zhu [15] reported a GC-MS method for AHL detection and revealed three AHLs from the extract of two strains isolated from refrigerated shrimp.

*Aeromonas sobria*, a Gram-negative, motile, flagellated, facultative anaerobic bacterial, is an opportunistic pathogen of animals, aquatics, and humans [16,17,18,19]. In recent years, research on QS in *Aeromonas* spp. has concentrated on *Aeromonas hydrophilia* and QS regulation of biofilm formation, virulence factors, protease activity and motility [20,21,22].

In this study, gas chromatography-mass spectrometry (GC/MS) was utilized to detect AHLs produced by *A. sobria* strain AS7, which was isolated from vacuum-packed, refrigerated putrid turbot. Meanwhile, the effect of environmental conditions on the production of AHLs, as well as AHL-production kinetics was studied by agar well diffusion assay. Furthermore, the exogenous autoinducers were added to the culture medium of AS7 and inoculated AS7 to sterile fish fillets. The purpose of the present work was to elucidate whether bacterial signals (AHLs) and AHL-dependent regulation contribute to the spoilage of fish.

## 2. Materials and Methods

### 2.1. Materials and Bacterial Strains

Live turbot were collected from a local aquatic products market (Jinzhou, China) and transferred to the laboratory with oxygenated water. The fish were killed and rinsed with sterile water. Then, the sample was vacuum-packed and refrigerated at −2 °C for 28 days. *N*-butanoyl-dl-homoserine lactone (C_4_–HSL), *N*-hexanoyl-dl-homoserine lactone (C_6_–HSL), *N*-octanoyl-dl-homoserine lactone (C_8_–HSL), *N*-decanoyl-dl-homoserine lactone (C_10_–HSL), *N*-dodecanoyl-dl-homoserine lactone (C_12_–HSL), *N*-tetradecanoyl-dl-homoserine lactone (C_14_–HSL) were purchased from Sigma-Aldrich (Poole, UK). Other reagents used in this study were commercially available and of analytical grade. *Chromobacterium violaceum* CV026 and *Agrobacterium tumefaciens* A136 were stored at our laboratory.

### 2.2. Isolation and Identification of Bacterial Strains

Ten grams of the samples were mixed with 90 mL sterile normal saline and flapped in BagMixer (Interscience, St. Nom, France) for 1 min. Then, the treated sample was continuously diluted and counted on Plate Count Agar (PCA, Aoboxing Bio-Tech, Beijing, China) and incubated at 28 °C for 48 h. After 48 h, all obviously distinct bacterial colonies were appraised and each colony was streaked onto *Aeromonas* Selective Medium Base (Ryan) followed by incubation at 28 °C for 24 h. The color, outline, size and other optical properties of the colonies were observed and recorded. The VITEK-2 Compact system (BioMerieux, Marcy l’Etoile, France) was used to identify the single colony [23]. Further study on the colony was validated with 16S rRNA PCR using the forward primer 27F (5′-AGAGTTTGATCCTGGCTCAG-3′) and the reverse primer 1492R (5′-ACGGCTACCTTGTTACGACTT-3′) amplify the 16S rRNA gene [24]. The gene sequences were analyzed by comparing the closely related sequences by BLASTN program in the GenBank database and phylogenetic analyses using MEGA 5.0 software.

### 2.3. AHL Detection of the Isolated Strain

The AHL molecule side chain length varies from short chain (C4) to long chain (C18) carbon chains. The strain to be tested for reaction of *C. violaceum* CV026 with the LuxR homologue, CviR, which regulates the production of a purple pigment when induced by particular AHLs, was parallel streaked on LB agar plate, which rapidly screened for short chain AHL production [13]. The presence of long chained AHLs detected by *A. tumefaciens* A136, which carries a *lac*Z fusion to *tra*I and produces a blue color in the presence of 5-bromo-4-chloro-3-indoyl-β-d-galactopyranoside (X-Gal), was done in a similar method supplementing with 20 μL X-Gal (20 mg/mL) [12]. C_4_–HSL and C_6_–HSL were used as positive controls for CV026 and A136, respectively. Negative controls were the monitor strains themselves.

### 2.4. Extraction of AHLs

In addition, 100 mL culture was centrifuged at 10,000 rpm for 10 min then extracted with an equivalent volume of ethyl acetate (0.1% (v/v) glacial acetic acid). The mixture was shaken adequately for 30 s and stayed for layer. This process was repeated three times before the ethyl acetate was removed and another 100 mL ethyl acetate was added. Then, the whole extraction process was repeated three times and the whole ethyl acetate fraction was mixed. The combined ethyl acetate fractions were evaporated with rotary evaporators (35 °C, 150 rpm) to dryness and redissolved in 1 mL methyl alcohol. The extracts were stored in sterile microcentrifuge tubes at −20 °C.

### 2.5. AHL-Production Kinetics

The value of AHLs was determined by the agar diffusion method using CV026. A preculture was done in LB medium and grown for 12 h at 28 °C, and 100 μL was diluted to 100 mL of LB medium. The culture was grown at 28 °C 160 rpm and was monitored by OD_600_ determinations. In addition, a 100 mL sample was withdrawn every four hours and centrifuged at 10,000 rpm for 10 min. The supernatant was used to analyze AHL-content in the well-diffusion assay [25]. Each well was supplemented with 200 μL supernatant and was grown for 48 h at 28 °C. Diameters of induced zones were measured and recorded.

### 2.6. Effect of Several Conditions on AHL Production in AS7

The effect of the carbon source on AHL production was studied in AB medium [26] supplemented with casamino acids (CAA, 0.5%) and 0.5% of one of the following carbon sources: glucose, sucrose, fructose, xylose, lactose or maltose. The influence of pH value was studied by Trypticase Soy Broth (TSB) media to pH 4, 5, 6, 7, 8 or 9 by mixing different volumes of phosphate buffer. The effect on AHL production by altering salt concentrations was studied in LB medium with 0.5%, 0.7%, 1.0%, 2.0%, 3.0%, 4.0% or 5.0% NaCl. Pre-cultures for experiments carried out at 28 °C were grown in TSB medium for 12 h and were diluted as 1:1000 to inoculate into various media. They were grown for 24 h at 28 °C 160 rpm and determined OD_600_. In addition, 100 mL solution was centrifuged at 10,000 rpm for 10 min and the supernatant was stored at −20 °C.

### 2.7. AHL Identification via Gas Chromatography-Mass Spectrometry (GC-MS)

Analyses were performed using a GC-MS Agilent 7890N/5975 (Agilent, Palo Alto, CA, USA) according to Zhu [15] with some modifications. All sample injections were done in the split mode (50:1) into an HP-5 MS capillary column (30 m length × 0.25 mm internal diameter × 0.25 μm film thickness) (Agilent, Palo Alto, CA, USA). Helium was used as the carrier gas at a flow rate of 1 mL/min. The GC injector temperature was 200 °C and the oven temperature was programmed as follows: 150 °C ramped at 10 °C/min to 220 °C, and ramped at 5 °C/min to 250 °C, then ramped at 0.5 °C/min to 252.5 °C. Mass spectrometry conditions were as follows: electron ionization source was set to 70 eV, MS Quad 150 °C, emission current 500 μA, MS Source 230 °C. Data were acquired by either full-scan mode (*m/z* 35–800) and in selected ion monitoring (SIM) mode (*m/z* 143).

### 2.8. Siderophore Assay

Siderophore production was detected on solid media using Chrome-Azurol-S CAS-agar [27] (NC Pharmculture CO., Ltd, Beijing, China) with some modifications. To prepare 1 L of blue agar, 60.50 mg CAS was dissolved in 50 mL distilled water and mixed with 10 mL iron(III) solution (1 mM FeCl_3_·6H_2_O, 10 mM HCl). Under mixing this solution was slowly added to 72.90 mg hexadecyl trimethyl ammonium bromide (HDTMA) dissolved in 40 mL distilled water. The composite dark blue liquid was autoclaved at 121 °C, 15 min, which was keeping the standby as the CAS assay solution. Also autoclaved was 30 mL casamino acids (10%), after cooling to 50 °C, stirring with 1 mL CaCl_2_ (1 mM), 20 mL MgSO_4_·7H_2_O (1 mM), 10 mL glucose (20%) as a carbon source, 15.00 g agar, 30.24 g 1,4-Piperazinediethanesulfonic acid (Pipes), and 12.00 g of a 50% (*w*/*w*) NaOH solution to raise the pH to the pKa of Pipes (6.8). Then the cultures were heated to 121 °C and maintained 15 min by autoclaved. After cooling to 60 °C, stirring the CAS assay solution mentioned above, the solution was slowly added by edgeways conical flask, with enough agitation without generation of foam. Each plate contained 20 mL of blue agar with punching by Oxford cup (autoclaved). These blue agars were used to detect siderophores. Exogenous autoinducers were added as follows: C_6_–HSL, 10, 20 and 40 μM; C_8_–HSL, 10, 20 and 40 μM. An equal volume of methyl alcohol was used as the negative control.

### 2.9. Fish Spoilage Assays

Sterile turbot fillets were made according to Herbert [28] with some modifications. Live turbot were killed and the fish fillets were washed by distilled water and drained off in the Clean Bench (Shiwei Ke Environmental Science and Technology Co., Ltd, Suzhou, China), then sprayed with 75% alcohol on the surface and skimmed over the outer flame of alcohol lamp. In order to confirm the fish fillets were sterile, samples were cultured in PCA to detect the total viable counts below 2.0 log cfu/g.

Prior to inoculation of fish, the strain AS7 was inoculated in 10 mL TSB and incubated overnight at 28 °C with shaking at 160 rpm. Then, the cultures were centrifuged at 10,000 rpm for 10 min, washed, and resuspended in sterile 0.85% physiological saline to produce a final optical density at 600 nm (OD_600_) of 1.0 [20]. Exogenous autoinducers were added as follows: C_4_–HSL, C_6_–HSL and C_8_–HSL 20 μM, respectively. An equal volume of methyl alcohol was used as the negative control. The sterile fish fillets were dipped in the bacterial suspension for 2 s, then packaged with sterile PVC bag at 4 °C.

## 3. Results and Discussion

### 3.1. Identification of Bacteria Isolates

**Figure 1:**
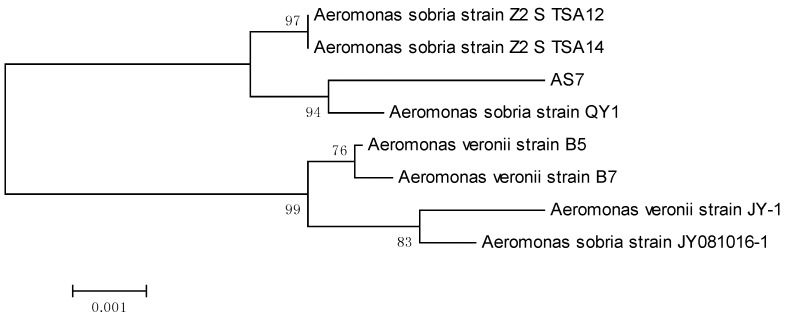
The phylogenetic relationships of strain AS7 and other *Aeromona*.

A pure strain AS7 was obtained after several successive streakings. Physically, the strain AS7 colonies were round, the surface wet, uplifted, transparent, edge neat little colony, and Gram-negative. Strain AS7 was identified using the Vitek-2 Compact system (bioMerieux, Marcy l’Etoile, France), which showed 99% similarity to *Aeromonas sobria*. Physiological and biochemical characters of strain AS7 were described in detail (Table 1). Strain AS7 was validated with 16S rRNA gene nucleotide analysis for further study, and the result was based on GenBank database and phylogenetic analysis of the 16S rRNA on MEGA (Figure 1), where the evolutionary history was deduced using the Neighbor-Joining method [29]. The percentage of repeat trees in which the related taxa clustered together in the bootstrap test (1000 repeats) was indicated next to the branches [30]. The evolutionary distances were calculated using the Maximum Composite Likelihood method [31] and were in the units of the number of base replacements per site. Our phylogenetic analysis results revealed that strain AS7 belonged to *Aeromonas sobria*.

### 3.2. Detection of AHLs

**Figure 2:**
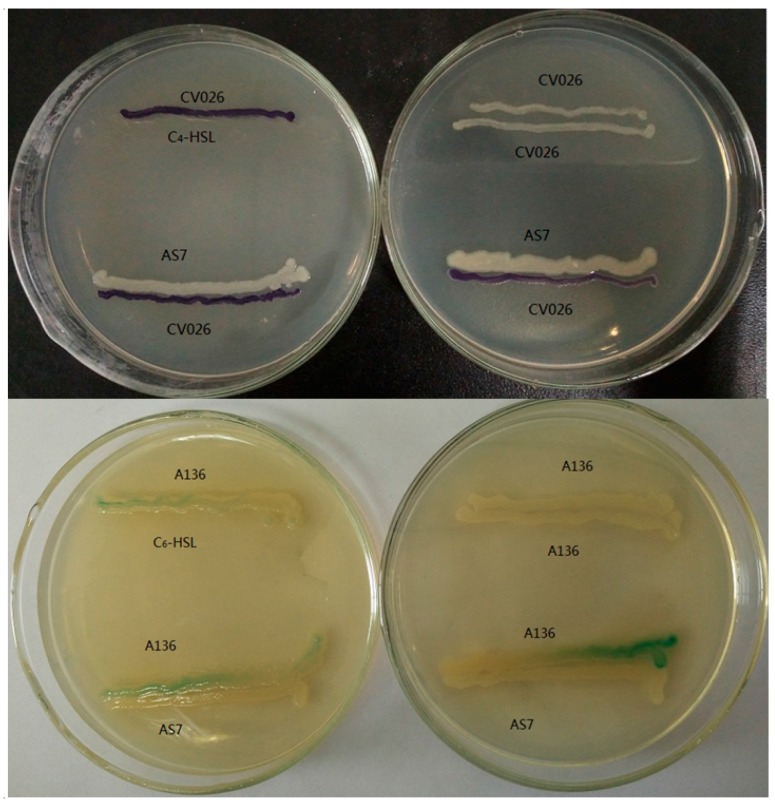
Streak assays for the production of short chain (*N*-acyl homoserine lactones (AHLs)) in test strain (screening for AHLs production using *C. violaceum* CV026 and *A.* *tumefaciens* A136 cross parallel streaking with C_4_–HSL (C_4_-homoserine lactones) and C_6_–HSL as positive control, respectively. Negative controls were the monitor strains themselves.).

AHLs produced by *A. sobria* strain AS7 were detected by using *A.* *tumefaciens* A136 and *C. violaceum* CV026. *A.* *tumefaciens* A136 carries a plasmid with P*traI-lac*Z fusion and produces a blue color by degrading the X-Gal in response to long chained AHLs [12,32]. The CviR of *C. violaceum* CV026 regulates the production of violacein when induced by short chained AHLs [12,33]. Strain AS7 showed positive results by inducing a blue color and violacein production in *A.* *tumefaciens* A136 and *C. Violaceum* CV026, respectively, suggesting both short and long-chained AHLs are produced by AS7 (Figure 2).

### 3.3. Short Chain AHL Production Kinetics

**Figure 3:**
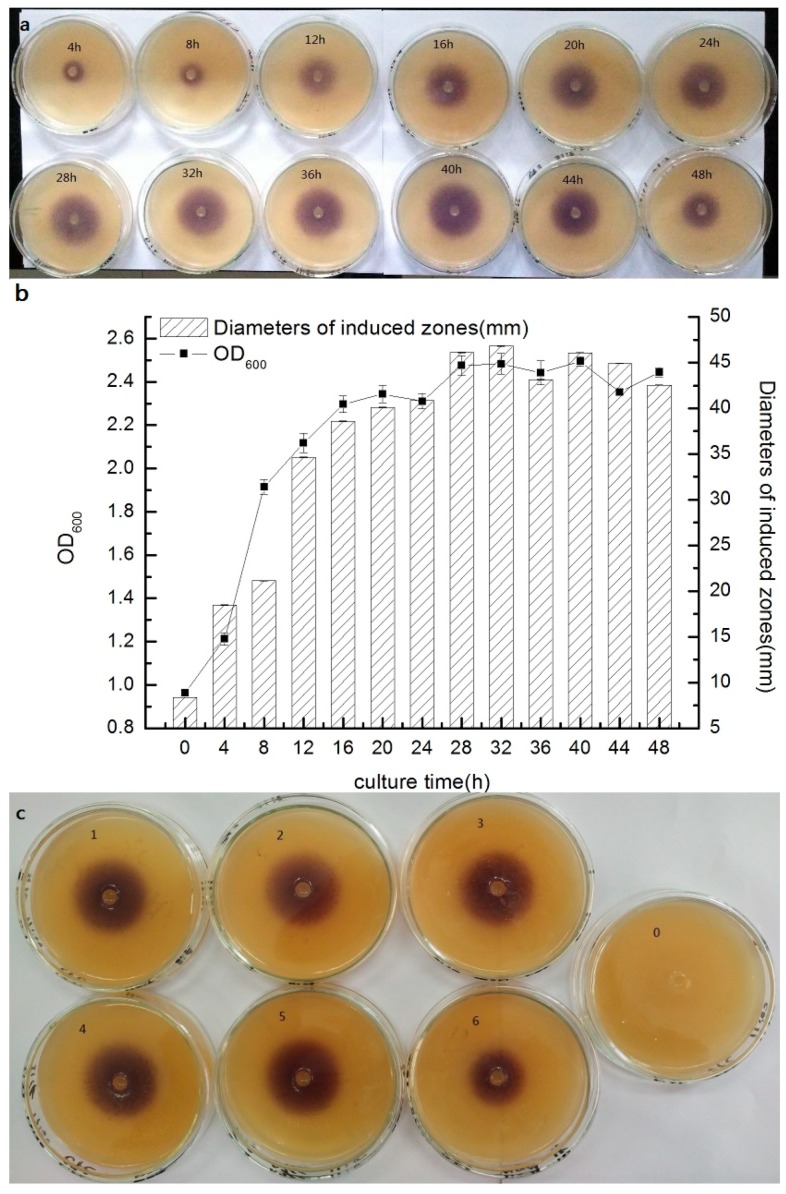

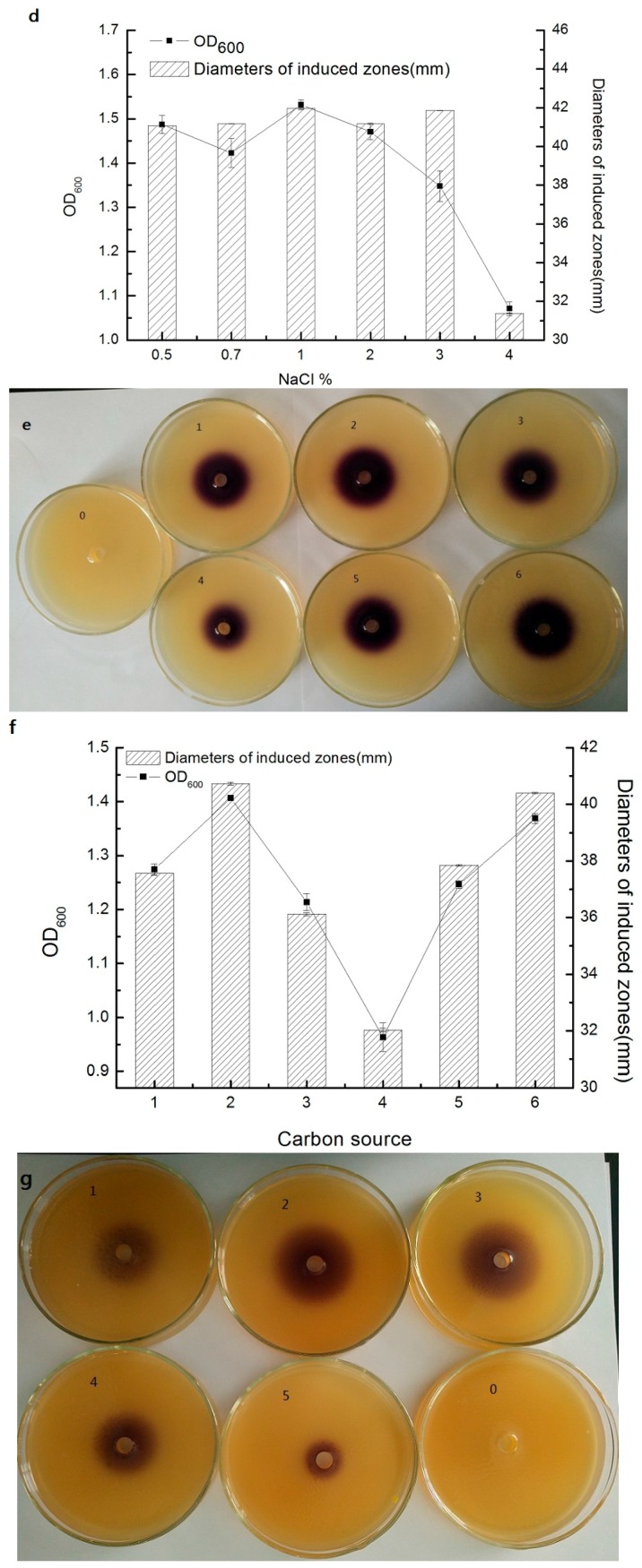

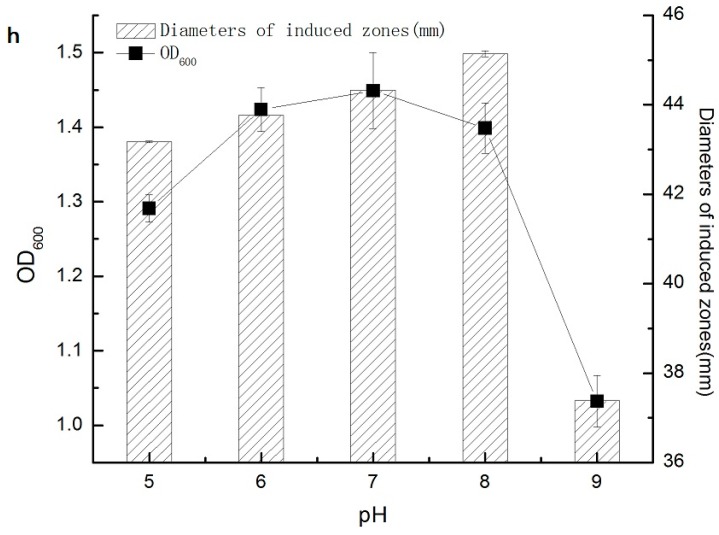
Relationship between growth kinetics and short chain AHL secretion under different culture time of strain AS7 and effect of different conditions on short chain AHLs production by AS7 (**a**): Agar well diffusion assay to detect AHLs secretion under different culture time; (**b**): the graph of relationship between kinetics and AHLs; (**c**,**d**): NaCl concentration 0: blank control, 1: NaCl 0.5%, 2: NaCl 0.7%, 3: NaCl 1%, 4: NaCl 2%, 5: NaCl 3%, 6: NaCl 4%; (**e**,**f**): carbon source 0: blank control, 1: glucose, 2: sucrose, 3: frutose, 4: xylose, 5: lactose, 6: maltose; (**g**,**h**): 0: blank control, 1: pH 5, 2: pH 6, 3: pH 7, 4: pH 8, 5: pH 9.

Short chain AHL-production was measured through a well-diffusion assay using *C. violaceum* CV026 as the monitor strain and the concentration of short chain AHLs in the supernatant was estimated by measuring the diameter of the induced zone. Strain AS7 has a stronger ability to secrete AHLs from 12 h to 44 h, and the concentration of AHLs was at a high level during this period (Figure 3a). At logarithmic phase of strain AS7 (4–16 h), the concentration increased rapidly as synchronous as the content of short chain AHLs-signal molecules (Figure 3b). Then, the stationary phase came and the content of short chain AHLs maintained a high level, and the concentration of signal molecules achieved the highest level at 32 h and was in accordance with bacterial density. The production of short chain AHLs showed a decrease trend after the initial increase with the extension of incubation time and presented the density dependence. This might be that the bacteria began to enter the decline period.

### 3.4. Effect of Different Conditions on Short Chain AHL Production by Strain AS7

Figure 3c showed the results for AHL production by strain AS7 under different contents of sodium chloride. In general, the results presented a prominent relationship between population levels and AHL production (Figure 3d). Our data was in accordance with previous reports about AHL QS systems in *Aeromonas* spp. [34]. At 4% (w/v) NaCl, the concentration of AHLs showed a significant decline compared to the other concentrations, which was correlated with a low colony count. When the concentration of NaCl was at 5%, the strain AS7 could not grow up. This might be that the high concentration of NaCl inhibited the growth of strain AS7. However, at low concentrations (0.5%–2%), the production of AHLs was at a high level and showed no obvious change. The production of signal molecules was about the maximum at 1% NaCl. This was probably because salinity controlled the production of AHLs. Jahid [8] reported that salinity could influence C_4_–HSL, C_6_–HSL, as well as biofilms, exoproteases and motility of *A. hydrophila* isolated from surface water. Medina-Martinez [34] reported that 3% NaCl completely inhibited AHL production of *A. hydrophila* isolated from food samples, which was accordance with our results.

The effect of different carbon source on short chain AHL production for strain AS7 was shown in Figure 3e. The results showed a significant relationship between the concentration of strain AS7 and the production of signal molecules (Figure 3f). The ability of carbon sources to influence the secreted AHLs of strain AS7 was as follows: sucrose > maltose > glucose > lactose > fructose > xylose. Sucrose was the best carbon source for the growth of strain AS7 and xylose was not conducive to the production of AHLs. Flodgaard [35] reported no effect on 3-oxo–C_6_–HSL secretion of *S. proteamaculans* by change of carbon sources. However, the growing environments of different species were variant. Strain AS7 could not make full use of xylose to increase the density and then influence the secretion of AHLs, which was consistent with our results that strain AS7 could use sucrose, maltose and glucose, but xylose was negative (Table 1).

With regard to AHL production at different pH values, AHL secretion was correlated with total viable count (Figure 3g,h). At pH 8, the concentration of AHLs was at the highest level; at pH 4, strain AS7 could not survive. Acid conditions (pH 5 and 6) and neutral environment were not significant to the production of AHLs. Acid conditions were lower than the neutral environment and the lower the pH was, the lower the AHL production. This might be that an adverse environmental condition (weak acid condition) could not lead to the effective growth of AS7, while at pH 9, a sharp drop of signal molecules was presented. Our data agreed with previous studies that AHLs were unstable at alkaline conditions [35,36].

### 3.5. GC-MS Analysis of AHLs in Extracts of Bacterial Cultures

**Figure 4:**
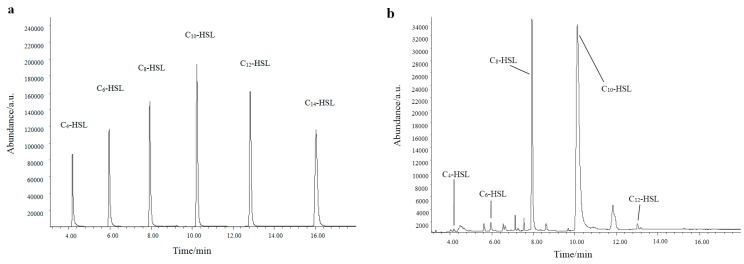
GC-MS chromatogram in SIM mode at *m/z* 143 of a standard mixture of AHLs (**a**) and an extract of cell-free supernatant of AS7 (**b**).

The GC-MS method was used to further verify the AHL production. All AHLs are characterized by a homoserine lactone moiety and a fatty acyl group whose members have various lengths, ranging from 4 to 14 carbons. The fragments of AHL standards were listed in Table 2. The ion at *m*/*z* 143 was chosen as the prominent fragment to detect extracted samples [37]. Meanwhile, a standard mixture of AHLs was detected and the retention times of C_4_–HSL, C_6_–HSL, C_8_–HSL, C_10_–HSL, C_12_–HSL, and C_14_–HSL were 4.172 min, 5.956 min, 7.942 min, 10.239 min, 12.836 min, and 16.044 min, respectively (Figure 4a). According to retention time and prominent fragments (*m/z* 143), C_4_–HSL, C_6_–HSL, C_8_–HSL, C_10_–HSL and C_12_–HSL were present in the extracted samples (Figure 4b). The detection of AHLs, peculiarly C_6_–HSL, was in agreement with reported studies as the AHL QS in the *Aeromonas* species that significantly produced C_6_–HSL [38]. Cataldi [37] demonstrated that *Aeromonas hydrophila* and *Aeromonas salmonicida* synthesized C_8_–HSL, C_12_–HSL, C_14_–HSL and C_8_–HSL, C_10_–HSL, C_12_–HSL, C_14_–HSL as major AHLs, respectively. Our results suggested that strain AS7 could produce five types of AHLs, with C_8_–HSL and C_10_–HSL being the major AHLs in particular. Although *Aeromonas* spp., such as, *Aeromonas hydrophila* and *Aeromonas salmonicida*, were studied extensively, the research on the types of AHLs secreted by *Aeromonas sobria* was reported firstly in detail.

### 3.6. The Regulation of Siderophore Secretion by QS in AS7

**Figure 5:**
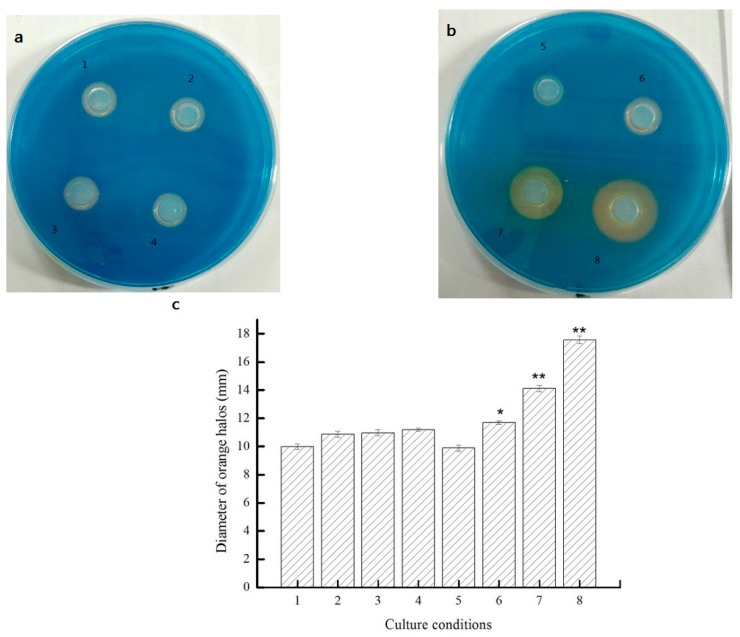
The effects of exogenous autoinducers on siderophore formation in AS7 (**c**)—(**a**): (**2**), 10 μM C_6_–HSL; (**3**), 20 μM C_6_–HSL; (**4**), 40 μM C_6_–HSL and (**b**): (**6**), 10 μM C_8_–HSL; (**7**), 20 μM C_8_–HSL; (**8**), 40 μM C_8_–HSL. (**1**) and (**5**) was as the control. Data were presented as the mean ± standard deviation (*n* = 3; * *p* < 0.05; ** *p* < 0.01).

Levels of siderophore production were tested on CAS-plates. Siderophores are small, iron-chelating molecules secreted by microorganisms to scavenge iron [39]. The color of the CAS complex, ferric iron and hexadecyltrimethylammonium (HDTMA), changed from blue to orange when iron was removed by siderophores. The diameters of orange halos were measured to determine the relative amount of siderophores produced [40]. Iron is essential for the growth of most microorganisms and is used in bacterial respiration (as electron shuttler) and in redox enzymes. Due to the high oxidative power of Fe^3+^, iron is mostly bound in insoluble complexes in the environment and in mammals and plants. Most microorganisms have therefore developed highly specific iron chelating systems, and they often produce siderophores, which are iron chelators secreted by the cell. All facultative anaerobic and aerobic bacteria require iron for growth. In order to compete, siderophores are produced under iron-limiting conditions by some bacteria. The production of siderophore is conducive for bacteria to maintain a high level density and have a population-wide benefit. As shown in Figure 5a, C_6_–HSL exerted no significant effect on the content of siderophores. However, C_8_–HSL significantly increased the production of siderophores (Figure 5b), meanwhile having an obvious correlation between the production of siderophores and the additive amount of C_8_–HSL (*p* < 0.01) (Figure 5c). C_8_–HSL could regulate the production of siderophores and present positive correlation in strain AS7. These results were in accordance with our previous study about the AHLs of strain AS7, which showed that C_8_–HSL was the maximum in extracted samples.

### 3.7. The Effect of QS on the Spoilage Process

**Figure 6:**
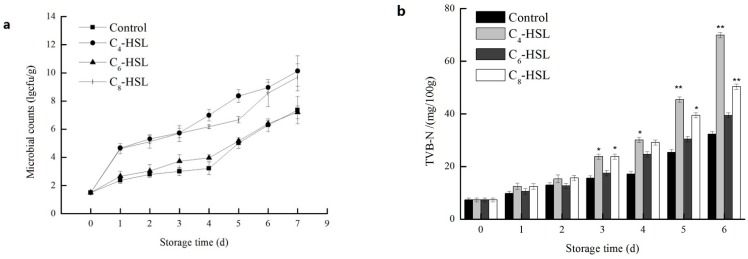
The effects of C_4_–HSL (20 μM), C_6_–HSL (20 μM) and C_8_–HSL (20 μM) on (**a**) microbial counts; and (**b**) TVB-N production in turbot blocks stored at 4 °C. Data were presented as the mean ± standard deviation (*n* = 6; * *p* < 0.05; ** *p* < 0.01).

Levels of total viable counts and TVB-N values are important quality parameters for evaluating the process of fish spoilage. As shown in Figure 6a,b, exogenous C_4_–HSL and C_8_–HSL significantly stimulated the production of TVB-N (*p* < 0.01), while no significant effect of exogenous C_6_–HSL was observed (*p* > 0.05). Total viable counts showed the similar results with TVB-N. Exogenous C_6_–HSL presented no significant effect on the fish fillet spoilage (*p* > 0.05). Bruhn [41] reported that AHLs did not influence the spoilage of vacuum-packed meat. The specific signaling molecule of AHLs might not the key factor of regulation, such as C_6_–HSL for strain AS7, while C_4_–HSL and C_8_–HSL enhanced total viable counts of fish-related bacteria in vitro. C_4_–HSL and C_8_–HSL regulated the secretion of proteases [15], the proteins were rapidly decomposed by the proteases and produced a putrid odor. Zhang [42] reported that addition of exogenous AHLs and QSI decreased the specific protease activity both of the *Serratia* A2 and *Aeromonas* B1 and exogenous AHLs enhanced the biofilm formation in *Aeromonas* B1. The effects of QS signaling molecules on the growth kinetics of spoilage in foods had been reported previously. Christensen [43] reported that *N*-(β-ketocaproyl)-l-homoserine lactone regulated the production of proteases. The different effects of C_4_–HSL, C_6_–HSL, and C_8_–HSL on TVB-N production and total viable counts corresponded with those on the growth of strain AS7, which confirmed the key role of the QS in the spoilage of turbot.

## 4. Conclusions

In conclusion, *A. sobria* strain AS7 showed QS activity with the production of C_4_–HSL, C_6_–HSL, C_8_–HSL, C_10_–HSL and C_12_–HSL according to GC-MS analysis. Meanwhile, the kinetics of AHL production were performed in our laboratory at present. The influences of different pH, NaCl concentrations and carbon sources on the production of AHLs were also studied. Siderophore production of AS7 could chelate the iron from the environment and build an environment of low iron, which could restrain other microbial growth, then make it into specific spoilage organisms (SSO) and accelerated spoilage. QS was involved in the spoilage of turbot. Our future studies will concentrate on the quantities of AHLs produced in response to changes in the environment, and the actual environment in foods should be responsible for stimulation and inhibition of AHLs production. There might be significance for the food industry to control food spoilage using an anti-QS strategy.

## Figures and Tables

**Table 1:** Physiological and biochemical characters of the AS7 strains.

Item	Phenotype	Item	Phenotype	Item	Phenotype
APPA	+	PyrA	−	dCEL	−
H_2_S	−	AGLTp	−	GGT	−
BGLU	−	dMAN	+	BXYL	−
ProA	−	PLE	−	URE	−
SAC	+	dTRE	+	MNT	−
ILATk	−	SUCT	+	AGAL	−
GlyA	−	LDC	−	CMT	+
O129R	+	IMLTa	+	ILATa	−
ADO	−	IARL	−	BGAL	+
BNAG	+	dGLU	+	OFF	+
dMAL	+	dMNE	+	BAIap	−
LIP	−	TyrA	+	dSOR	−
dTAG	−	CIT	−	5KG	−
AGLU	−	NAGA	−	PHOS	−
ODC	−	IHISa	−	BGUR	−
GGAA	+	ELLM	+		

Notes: “+” for positive, “−“ for negative.

**Table 2:** The SIM parameter of six kinds of AHLs.

AHLs	Fragment	Retention Times/min
C_4_–HSL	32,43.1,57.1,71.1,83,102.1,125.1,143,153.1,171.1	4.172
C_6_–HSL	32,43.1,56.1,71.1,83,99.1,102.1,125,143.1,156.1	5.956
C_8_–HSL	32,43.1,57.1,69.1,83.1,102.1,125.1,143.1,156.1,207	7.942
C_10_–HSL	32,43.1,57.1,69,83.1,102.1,125,143,156.1,207	10.239
C_12_–HSL	32,43.1,57.1,71.1,83,102.1,125.1,143,156.1,207	12.836
C_14_–HSL	32,43.1,57.1,69,83.1,102.1,125.1,143,157.1	16.044

## Abbreviations

The following abbreviations are used in this manuscript:

APPA	Ala-Phe-Pro-ARYLAMIDASE
H2S	H2S PRODUCTION
BGLU	BETA-GLUCOSIDASE
ProA	l-Proline ARYLAMIDASE
SAC	SACCHAROSE/SUCROSE
ILATk	l-LACTATE alkalinisation
GlyA	Glycine ARYLAMIDASE
O129R	O/129 RESISTANCE (comp.vibrio.)
ADO	ADONITOL
BNAG	BETA-*N*-ACETYL-GLUCOSAMINIDASE
dMAL	d-MALTOSE
LIP	LIPASE
dTAG	d-TAGATOSE
AGLU	ALPHA-GLUCOSIDASE
ODC	ORNITHINE DECARBOXYLASE
GGAA	Glu-Gly-Arg-ARYLAMIDASE
PyrA	l-Pyrrolydonyl-ARYLAMIDASE
AGLTp	Glutamyl Arylamidase pNA
dMAN	d-MANNITOL
PLE	PALATINOSE
dTRE	d-TREHALOSE
SUCT	SUCCINATE alkalinisation
LDC	LYSINE DECARBOXYLASE
IMLTa	l-MALATE assimilation
IARL	l-ARABITOL
dGLU	d-GLUCOSE
dMNE	d-MANNOSE
TyrA	Tyrosine ARYLAMIDASE
CIT	CITRATE (SODIUM)
NAGA	Beta-*N*-ACETYL-GALACTOSAMINIDASE
IHISa	l-HISTIDINE assimilation
ELLM	ELLMAN
dCEL	d-CELLOBIOSE
GGT	GAMMA-GLUTAMYL-TRANSFERASE
BXYL	BETA-XYLOSIDASE
URE	UREASE
MNT	MALONATE
AGAL	ALPHA-GALACTOSIDASE
CMT	COURMARATE
ILATa	l-LACTATE assimilation
BGAL	BETA-GALACTOSIDASE
OFF	FERMENTATION/GLUCOSE
BAIap	BETA-Alanine arylamidase pNA
dSOR	d-SORBITOL
5KG	5-KETO-d-GLUCONATE
PHOS	PHOSPHATASE
BGUR	BETA-GLUCORONIDASE
